# Microbial fuel cell technology for clean energy production from palm oil mills effluent liquid waste

**DOI:** 10.1039/d5ra10122k

**Published:** 2026-04-13

**Authors:** Rinna Juwita, Meilana Dharma Putra, Chairul Irawan, Abubakar Tuhuloula, Zainuddin, Rosida Rizkya, Indah Retno Wulandary, Farid Fadhillah

**Affiliations:** a Department of Chemical Engineering, Lambung Mangkurat University Banjarbaru 70714 Indonesia mdputra@ulm.ac.id; b Department of Chemical Engineering, Imam Mohammad Ibn Saud Islamic University (IMSIU) Riyadh 11432 Saudi Arabia

## Abstract

The microbial fuel cell (MFC) technology utilizes microbial metabolism for energy conversion. This study uses palm oil mill effluent (POME) as a substrate, the effective microorganism (EM4), and *Escherichia coli* (*E. coli*) as microorganisms in a dual-chamber MFC system. This study aims to analyze the effect of bacteria on the electrical energy produced from the POME substrate, analyze changes in chemical oxygen demand (COD) and biological oxygen demand (BOD) in the waste, and analyze the effect of incubation time on the resulting power density value. pH, voltage (V), and electric current strength (mA) were observed at predefined time intervals over 36 h (0–36 h); while the biomass of *Escherichia coli* and EM4 was calculated accordingly. COD and BOD were tested before and after the microbial fuel cell (MFC) process. The maximum voltage and current values for *E. coli* occurred at the 26th hour, with a bacterial concentration of 25% (v/v), yielding 1.617 V and 0.844 mA, respectively, with a biomass amount of 0.3118 g mL^−1^ and a power density value of 174.075 mWatt m^−2^. However, the 20% (v/v) *E. coli* treatment resulted in the most pronounced reductions in BOD (57%) and COD (42%) among all tested conditions. The results further support the integration of microbial fuel cell technology into circular bioeconomy frameworks, where POME can be treated not only as waste but also as a resource for renewable energy generation.

## Abstract 2

Microbial fuel cells enabled the effective conversion of palm oil mill effluent into bioelectricity, accompanied by improved power output and significant reduction of organic pollutants.

## Graphical abstract

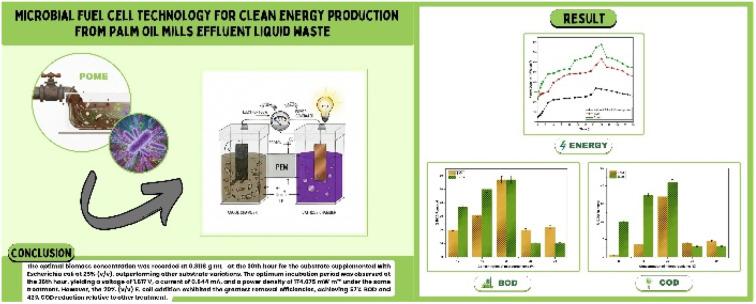

## Introduction

Palm oil is the most widely consumed vegetable oil worldwide and a raw material for chemicals and biofuels. The palm oil industry has emitted excessive waste materials, greenhouse gases, and an abundance of wastewater, and brought about land use change, affecting natural resources, despite the potential sustainability of the industry.^1^ The palm oil industry generates large volumes of palm oil mill effluent (POME). The management of this POME requires efficient technologies to reduce this unmanageable waste from spreading further.^2,3^ Although POME is not hazardous, throwing it away without first digesting can be extremely harmful because of its high levels of COD (Chemical Oxygen Demand) and BOD (Biological Oxygen Demand). Additionally, POME contains high levels of phosphorus (P) and nitrogen (N). Hence, it can be considered as a substrate for the growth of microorganisms.^4^

The pollutants in POME have harmful effects on water quality, aquatic life, soil, groundwater, and the environment. They must be effectively treated before disposal.^5,6^ Several established pre-processing methods, such as physical and chemical methods, adsorption, and coagulation–flocculation, can reduce pollutants in POME. However, each of these approaches has specific limitations that affect their overall effectiveness. These methods are effective in lowering suspended solids, oil and grease, and organic matter concentrations, although they are often associated with high chemical consumption and sludge generation.^7,8^ Given the limitations of current pretreatment techniques, there is a clear need for more effective methods to reduce pollutants in POME. This opens up opportunities for innovative treatment approaches and presents a significant challenge for POME management.^9^

The world's energy demand is rising quickly due to population expansion and industrial development. Fossil fuels are currently the primary source of energy for both industrial production and human use.^10^ However, the greenhouse effect and air pollution are caused by gaseous emissions from burning fossil fuels. Furthermore, a possible energy crisis will arise from the enormous use of fossil fuels.^11^ One promising substitute for conventional fossil fuels is the microbial fuel cell (MFC). It has massive potential promise for biomass valuation, waste management, and electricity generation.^12,13^ MFCs are a bioelectrochemical system that utilizes bacterial activity as a catalyst to convert biochemical reactions into electron flow so that it can produce electrical energy. A typical MFC comprises two chambers, an anode and a cathode that are separated by a proton exchange separator.^14^ A dual-chamber microbial fuel cell device has been successfully designed to generate bioelectricity from palm oil liquid waste.^15^ Among the various alternatives available to address pressing water and energy problems, the MFC is a noteworthy technology. It shows great potential for producing electricity and treating wastewater simultaneously.^16–18^ The important characteristics of MFC, such as simplicity of design, cost-effectiveness and low maintenance, are now widely recognized.^19^ The operation of MFCs generates bioelectricity, offering potential economic advantages.^20^

MFC offers a promising approach for converting organic waste into electricity. Because MFC-derived energy generation produces power from a variety of soluble wastes, it may be considered a potentially sustainable energy source for the future. The proper functioning of MFCs depends on many factors, including pH, temperature, conductivity, air supply to the cathode, anaerobic process at the anode, electron transport mediators, moisture, nutrient sources, and reactor design. In MFC systems, microbes obtain various substrates ranging from the simplest to the most complex.^21,22^ In rural or isolated locations that are not connected to the electrical grid, the MFC concept is one of the efficient methods for managing various wastes. It is also utilized to generate electricity to fulfill energy demands. MFCs lessen the strain on non-renewable energy resources and the global energy problem.^23^ The various biodegradable substrates used as fuel, coupled with the mild operating conditions of MFCs, have attracted the interest of researchers.^24^ A few studies have utilized real wastewater, such as dairy waste, textile waste, and palm oil mill effluent in MFCs to generate electricity.^4,25^

A wide range of pollutants and a variety of substrates were used to identify significant linkages for a large deal of potential through the deployment of MFC technology as a promising renewable energy source for the future.^26^ In a lab-based microalgae-microbial fuel cell (MMFC) system, the bioremediation of synthetic wastewater and the generation of bioelectricity were evaluated. The MMFC configuration with *D. subspicatus* at the cathode and *E. coli* at the anode showed the most effective performance in generating bioelectricity. The investigated systems were able to simultaneously clean wastewater and generate bioelectricity.^27^ A study successfully demonstrated the generation of bioelectricity from 100% concentrated palm oil mill effluent (POME) using a fuel cell. By employing aluminium, copper, and zinc electrodes and *Lactobacillus bulgaricus* as a biocatalyst, the researchers achieved a voltage of 1.52 Volts, a current of 3.35 mA, a power output of 5.092 mW, and an optimal power density of 0.127 mW cm^−2^.^28^ However, this study did not evaluate reductions in BOD and COD, even though both parameters are important for assessing the effectiveness of MFC as an energy producer and waste treatment technology. On the other hand, a dual chamber microbial fuel cell device has been successfully designed to generate bioelectricity from palm oil liquid waste. The addition of 10% *Lactobacillus bulgaricus* to palm oil liquid waste produced the best performance with a current of 0.9640 mA, a voltage of 0.6760 V, and a power density of 248.04 mW m^−2^. Furthermore, the MFC system could also reduce TSS (Total Suspended Solids) and COD (Chemical Oxygen Demand), with a decreasing rate of 42.6% and 7.2%, respectively.^15^ However, the use of *Lactobacillus bulgaricus* requires relatively expensive growth media and sensitive culture conditions, making it more challenging to develop than other microorganisms.^29^

A significant research void exists in the comprehensive characterization of microorganisms employed in MFC systems. This gap is particularly evident in the limited exploration of specific microbial variations, such as *Escherichia coli* and EM4. Further investigation is urgently needed because each microorganism has different characteristics that influence electroactive behavior, yet studies on this topic remain limited. Therefore, this study aims to analyze the effect of bacteria on electrical energy produced from the POME substrate, analyze changes in COD and BOD in the waste, and analyze the impact of incubation time on the resulting power density value. In this study, wastewater treatment performance was evaluated, including COD and BOD parameters, as well as an assessment of output voltage and power density for bioelectricity generation. This design is applicable to the palm oil mills effluent wastewater treatment plant. The results of this study provide opportunities for implementing wastewater treatment in the future by utilizing simpler designs and economical two-chamber MFC technology.

## Experimental

### Material and instruments

The tools used in this study include an MFC reactor consisting of dual chambers connected by alligator clip test leads equipped with a digital multimeter and a magnetic stirrer. The glassware used includes a measuring cup, glass beaker, Erlenmeyer flask, stove wick, stirrer glass, watch glass, dropper pipette, volume pipette, and pro-pipet. In addition, incubators, ovens, centrifuges and tubes, autoclaves, stove wicks, syringes, analytic digital, desiccators and gloves are also used.

### Substrate characterization

The raw palm oil mill effluent (POME) used in this study is a highly complex wastewater with significant variability in composition. The initial COD, BOD, and pH of the POME were 55 218.2 mg L^−1^; 13 830 mg L^−1^; and 5, respectively. Although oil and grease as well as total nitrogen and phosphorus contents were not directly measured in the present study, these parameters are known to contribute significantly to POME characteristics and are recommended for inclusion in future investigations to further enhance reproducibility and comparative assessment.

### Methods

#### Preparation

Initially, electrolysis equipment was prepared, including the proton exchange separator, the electrodes, and the electrolyte solution. The low cost-woven fabric used as the separator, was firstly boiled in a solution of 3% H_2_O_2_ for one hour, and then rinsed three times with distilled water. The separator was subsequently boiled again in 0.5 M H_2_SO_4_ for one hour, and then washed with distilled water three times. Before installation in the MFC reactor, the separator was soaked in distilled water and then dried.

Meanwhile, the electrodes were conditioned by soaking them in a 1 M HCl solution for 24 hours, followed by rinsing them with distilled water. After this step, the electrode was immersed in 1 M NaOH solution for the next 24 hours, and then rinsed again with distilled water. Finally, the pre-conditioned electrodes were soaked in distilled water until use. Potassium permanganate solution (KMnO_4_) was used for the electrolyte solution by dissolving 158 g of potassium permanganate in 1 liter of distilled water.

Potassium permanganate was specifically selected as the catholyte in this experiment to obtain a highly oxidative and kinetically favorable electron acceptor, thereby minimizing cathodic constraints and enabling a clearer assessment of the anodic microbial activity.^30^ The use of KMnO_4_ was intended to establish a controlled experimental environment in which the effects of different microbial inocula on electricity production could be reliably compared. Such an approach is commonly used in laboratory-scale MFC studies to decouple anodic behavior from cathode-related constraints.

#### Process

The next stage involved the MFC process, which begins with the preparation of the substrate. The substrate was obtained from the palm oil industry IV Regional V Pelaihari area, and incubated for two 24 hours periods. Bacterial culture was then prepared by growing a pure culture of *E. coli* in a Nutrient Broth (NB) containing 24 grams and 1% glucose in 1 liter of distilled water in an Erlenmeyer flask. The broth was filtered, subsequently sterilized in an autoclave at 121 °C for 15 minutes, and then cooled to 28 °C. Once cooled to this temperature, the pure bacterial culture was transferred to fresh medium using a sterile needle. The fresh medium was incubated at 30 °C for one day. For activation, 25 mL of EM4 was mixed with 500 mL of water and 1/8 tsp of liquid brown sugar, then fermented in a closed container for 2–4 days.

The dual-chamber MFC reactor consists of two 1 L compartments, with an anode and a cathode in each compartment. Both the anode and cathode ware carbon-based electrodes with the surface area of 7.84 × 10^−3^ m^2^. The electrode used was 7 cm × 5 cm × 0.35 cm. The substrate and inoculum were placed in the anode compartment, while the cathode chamber contained an electrolyte solution of KMnO_4_. The two chambers were separated by stove wick as a low-cost woven fabric separator, which was selected as an economical alternative to commercial Nafion membranes. The MFC was operated in batch mode, with a total operation time of approximately 36 h, corresponding to the hydraulic retention time. Electrical output was measured using a fixed external circuit without systematic variation of external resistance; therefore, polarization analysis was not conducted. These operating conditions are explicitly stated to ensure reproducibility and to allow meaningful comparison with related studies. Experiments were conducted by observing and measuring voltage (mV), current (mA), and pH every 2 hours over a 36 hours treatment period. Biomass measurements were used to calculate the dry weight of *E. coli* and EM4 cells every six hours over a 36 hours treatment period, and power density was calculated. BOD and COD content were tested before and after the MFC process.

**Fig. 1:**
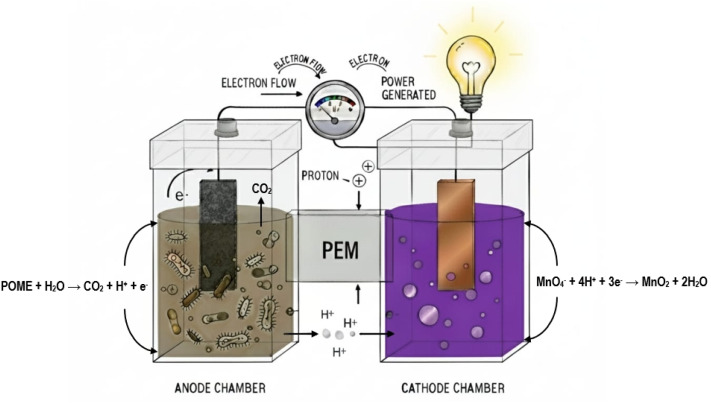
Schematic illustration of the microbial fuel cell (MFC) during POME treatment.

Fig. 1 illustrates the working principle and dominant metabolic and electrochemical pathways of the microbial fuel cell (MFC) system employed in this study. In the anode chamber, complex organic compounds present in palm oil mill effluent (POME) are oxidized through microbial metabolic processes, resulting in the release of electrons and protons. The electrons generated during substrate oxidation are transferred to the anode electrode and flow through the external circuit, producing electrical current, while protons migrate toward the cathode compartment through the separator. The overall anodic reaction can be represented in a generalized form as:

1 Organic matter (POME) + H_2_O → CO_2_ + H^+^ + e^−^

In the cathode chamber, potassium permanganate (KMnO_4_) serves as the terminal electron acceptor. Under acidic conditions, electrons arriving at the cathode reduce permanganate ions according to the reaction:

2 MnO_4_^−^ + 4H^+^ + 3e^−^ → MnO_2_ + 2H_2_O

The schematic highlights the coupling between microbial metabolism and electrochemical reactions, demonstrating how organic matter degradation in the anode is directly linked to electricity generation and cathodic reduction processes in the MFC system.

#### Experimental analysis

The biomass measurement procedure (dry weight method) was carried out by first weighing an empty centrifuge tube using an analytical balance to determine the initial weight (M1). Furthermore, a waste sample of up to 5 mL was taken from the microbial fuel cell (MFC) process using a volume pipette and placed in an empty centrifuge tube. The biomass in the centrifuge tube was dried in an oven at 80 °C for 1–1.5 hours and placed in a desiccator to cool. Afterwards, the tube was weighed using an analytical balance until the weight was constant (M2).

3 Biomass weight = (*M*_2_ − *M*_1_)/*V*

*M*_1_ = Weight of empty centrifuge tube (g).

*M*_2_ = Weight of centrifuge tube + weight of culture (g).

*V* = Volume (mL).

Coulombic efficiency (CE) was calculated to evaluate the fraction of electrons from organic matter oxidation recovered as electrical current, based on integration of the current–time profile and the corresponding COD removal in the anode compartment, following established literature methods.^31^

Chemical oxygen demand (COD) was determined using the closed reflux dichromate method in accordance with standard wastewater analysis procedures. Briefly, samples were digested with potassium dichromate under acidic conditions and the residual dichromate was quantified to determine COD values. Biochemical oxygen demand (BOD_5_) was measured using the 5 days incubation method, in which samples were incubated at 20 ± 1 °C under dark conditions, and the decrease in dissolved oxygen was recorded. BOD values were calculated from the difference between initial and final dissolved oxygen concentrations. All measurements were performed in accordance with established standard methods to ensure data reliability and reproducibility.

## Result and discussion

### Microorganism productivity in MFC

#### Kinetic profile of biomass

A microbial fuel cell (MFC) is a technology that utilizes microbes to generate electrical energy as primary outputs. This output is achieved as microorganisms act as the primary bio-activators, decomposing the waste used as the substrate. This study used *E. coli* and EM4 as bio-activators in POME waste. The amount of electrical power generated may increase or decrease depending on the microbial growth in the substrate which was observed through an increase in biomass value.

**Fig. 2:**
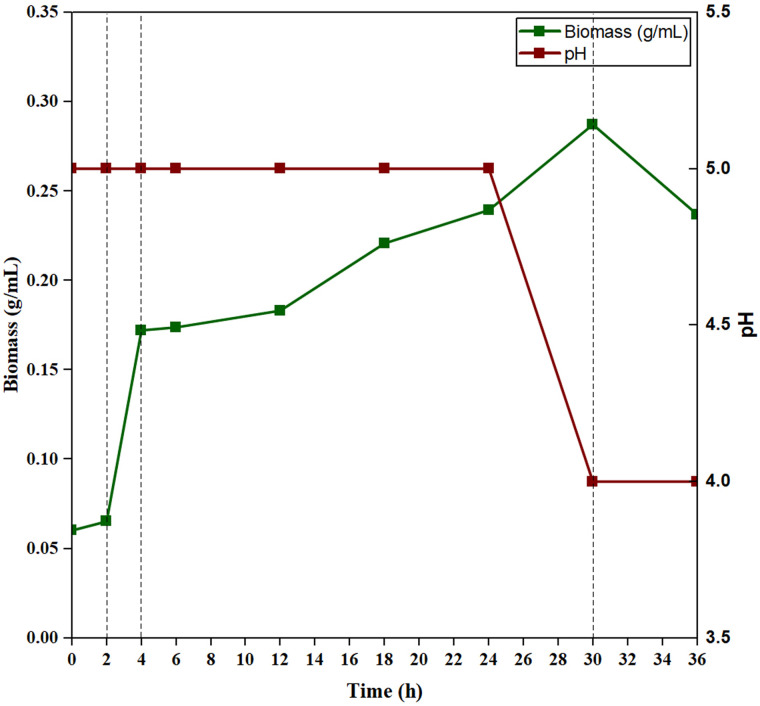
Effect of reaction time on biomass growth under the addition of 20% (v/v) *E. coli* (0–36 h, discrete sampling times).

Fig. 2 illustrates the increase in biomass over time during the MFC process with the addition of 20% (v/v) *E. coli* concentration. The graph depicts the phases of microbial growth throughout the study and also facilitates the identification of the optimum growth time. Changes in the slope of the curve signify transitions between microbial growth phases. During the lag phase of this investigation, which lasted from the 0^th^ to the 2nd hour, the microbial cells adapted to their new environment and began producing the cellular components required for growth.^28^ Diauxic growth, described by initial exponential growth followed by a phase of no growth, then continuing to a second exponential growth phase, takes place during the log phase. This occurs when microbes are given two sugars that can be metabolized.^32^

The optimum biomass was achieved at the 30th hour. The increase in biomass indicates that the microorganisms utilize the organic matter or carbon in the POME effluent and convert it into energy, thereby supporting their metabolic processes.^33^ The substrate condition at pH 5 is also responsible for the optimal increase, which can be due to the fact that bacteria grow most effectively at this pH.^34^ During the log phase of the growth cycle, power densities increased quickly. After reaching peak values, they started to diminish because of a decline in the amount of available organic matter for the microbes.^35^ The decrease in biomass occurred after 30 hours during the MFC process. This phenomenon indicates that the nutrient content in the substrate, as the food source for microbes, begins to decrease. Consequently, the number of microbes decreased and entered the death phase.^36^

This effect means that the nutrient content of the substrate, which is the main source of carbon and energy by which microorganisms are active, is gradually depleted as easily biodegradable organic matter is used up. To overcome this drawback and maintain microbial metabolism and electricity production, a number of operational strategies may be put into consideration such as continuous or fed-batch operation, periodic addition of easily biodegradable substrates, hydraulic retention time optimization, and staged or multi-chamber MFC design.^37^ The methods are capable of aiding to uphold substrate supply, lessen the metabolic inhibition and increase the stability of the system in the long term in the practical applications.

The acidogenic reactions that lead to the reduction of pH between 5 and 4 during MFC operation are mainly linked to the build-up of volatile fatty acids and release of protons during anodic oxidation.^38^ Although this acidification is typical of batch-operated MFCs treating complex organic wastewaters, the long-term proton buildup can adversely impact the microbial metabolic activity and elevate internal resistance. In practical terms, unregulated acidification might reduce the stability of operations in the long run and the ability to produce electricity. Scalable applications would thus require buffering mechanisms, *e.g.* phosphate buffer systems, pH-regulated operation or continuous-flow arrangement. These observations emphasize that pH control is a key operational parameter for sustaining long-term MFC performance, particularly when treating complex organic wastewaters such as POME.

In this study, biomass was measured by a dry weight technique, which reflects the total microbial biomass and does not differentiate between suspended cells and anodic biofilm. Although the anodic biofilm formation is a vital component of electricity production in MFC systems, direct morphologic characterization of biofilm by scanning electron microscopy (SEM) was not performed. Accordingly, the biomass data are interpreted as an indicator of overall microbial growth rather than a direct measure of electroactive biofilm growth. Further mechanistic understanding of biofilm formation and its role in electron transfer should be addressed in future research employing SEM and other surface-specific characterization techniques.

#### Microorganism productivity

Table 1 shows the microorganism productivity for both *E. coli* and EM4 at a concentration of 15%. As shown in Table 1, *E. coli* had a higher maximum biomass (0.2400 g mL^−1^) than EM4 (0.1680 g mL^−1^). This demonstrates *E. coli*'s ability to grow faster and adapt more effectively to organic substrates in POME, thus contributing significantly to the rapid degradation of organic compounds.^39^ In terms of specific growth rate, *E. coli* was superior (0.4332 h^−1^) to EM4 (0.3091 h^−1^), indicating a higher cell multiplication rate. This is important in the initial stages of waste because it accelerates the hydrolysis and acidogenesis phases, which determine the rate of waste degradation or COD/BOD reduction.^40^

**Table 1:** Microorganism productivity at different types of microorganisms

Micro-organism	Initial pH	Final pH	Microorganism productivity		
			Max. Biomass (g mL^−1^)	Max. Biomass productivity (g mL^−1^ h^−1^)	Max. Specific growth (h^−1^)
EM4	5	4	0.1680	0.0206	0.3091
*E. coli*	5	4	0.2400	0.0448	0.4332

*E. coli* showed a higher biomass productivity (0.0448 g mL^−1^ h^−1^) than EM4 (0.0206 g mL^−1^ h^−1^). This value is consistent with *E. coli*'s high specific growth rate (*µ*_max_ = 0.4332 h^−1^) and higher maximum biomass (0.2400 g mL^−1^). This combination confirms that *E. coli* is capable of converting organic substrates into biomass more quickly and efficiently. This is consistent with the character of *E. coli* as a facultative anaerobic bacterium that is capable of rapid growth and dominates the early phase of wastewater degradation.^39^ In contrast, EM4 only produced a maximum biomass of 0.1680 g mL^−1^ with lower biomass productivity and maximum specific growth rate of 0.0206 g mL^−1^ h^−1^ and 0.3091 h^−1^, respectively. Nevertheless, EM4 as a microbial consortium (lactic acid bacteria, yeast, photosynthetic) remains important in stabilizing long-term fermentation.^41^ The decrease in pH from 6 to 4 in both microorganisms indicates the formation of organic acids from the degradation process. This condition is common in the early stages of anaerobic degradation, but to maintain sustainability until the methanogenesis phase, a balanced microbial system is required.^42^

### Effect of *E. coli* concentration

Table 2 shows the effect of increasing *E. coli* concentration from 10 to 30% on the maximum biomass, maximum biomass productivity, and maximum specific growth rate. As shown in Table 2, increasing *E. coli* inoculum concentration from 10 to 25% positively impacts microbial productivity during POME treatment. This increase indicates a synergistic effect at higher inoculum concentrations, where a higher initial cell count accelerates the transition from the lag phase to the exponential phase and increases substrate utilization efficiency. This phenomenon is consistent with the principle of microbial kinetics that increasing inoculum concentration can accelerate the achievement of the logarithmic phase, thereby increasing substrate consumption.^43^

**Table 2:** Microorganism productivity at different *Escherichia coli* concentrations

*E*. *coli* concentration	Initial pH	Final pH	Microorganism productivity		
			Max. Biomass (g mL^−1^)	Max. Biomass productivity (g mL^−1^ h^−1^)	Max. Specific growth (h^−1^)
10%	5	4	0.2392	0.0380	0.3940
15%	5	4	0.2400	0.0448	0.4332
20%	5	4	0.2871	0.0535	0.4858
25%	5	4	0.3118	0.0707	0.4933
30%	5	4	0.3081	0.0647	0.4844

However, the increase in maximum biomass, maximum biomass productivity, and maximum specific growth rate from 20 to 25% was greater than that from 10 to 15%. A greater increase in this interval indicates an accelerated transition to the exponential phase and more efficient substrate utilization, which accelerates the decline in organic load in POME.^44,45^ These results are consistent with the principle that increasing the inoculum dose can accelerate the population's entry into the logarithmic phase and increase the rate of degradation of complex organic compounds.^39^ However, this increase also indicates that there is an optimal point, as too high a concentration can lead to substrate competition or the accumulation of inhibitory metabolites.^46,47^ On the other hand, too high a concentration (>20%) can cause the accumulation of organic acids, which lowers the pH and potentially inhibits methanogenic bacteria.^42,48^ Therefore, although 25% has been shown to yield the highest productivity, a long-term strategy still requires a balanced microbial community to maintain sustainability through the methanogenesis phase.

### Reduction of biological oxygen demand (BOD) and chemical oxygen demand (COD)

Organic compounds are among the primary pollutants contributing to water body contamination worldwide. Pollutants entering water sources alter the physicochemical characteristics of the water, placing stress on aquatic ecosystems or the water bodies themselves.^49^ Biochemical Oxygen Demand (BOD) and Chemical Oxygen Demand (COD) are the indicators used to assess wastewater pollution, as they reflect the concentration of degradable organic compounds present in the effluent.

**Fig. 3:**
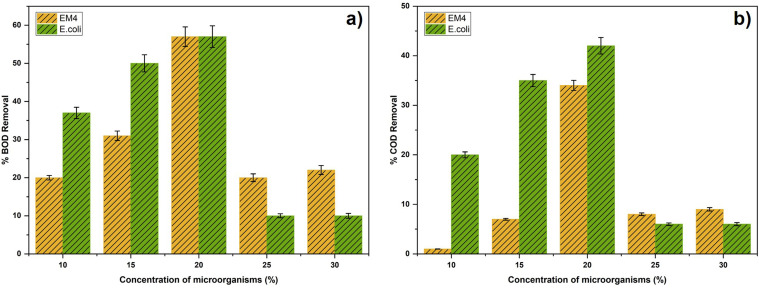
Effect of microorganism concentration on Biological Oxygen Demand (BOD) reduction (a) and Chemical Oxygen Demand (COD) reduction (b).

Fig. 3a demonstrates the effect of microorganism concentration on the reduction of Biochemical Oxygen Demand (BOD) across all variations. This reduction is attributed to the increased microbial population within the substrate, which enhances the breakdown of organic matter. The greater microbial activity leads to a more substantial decrease in BOD. This degradation process is referred to as biological decomposition. The decomposition of organic matter involves the breakdown of easily degradable organic compounds. As the process progresses, the oxygen demand decreases, resulting in a reduction in biochemical oxygen demand (BOD) of the substrate.^38^ The most significant percentage reduction in BOD among substrate variations was observed when *E. coli* and EM4 were added, reaching 57% compared to the control without microbial addition. Among different *E. coli* and EM4 concentrations, the most significant BOD reduction was recorded at 20%. This suggests that introducing higher concentrations of microbes into the substrate leads to greater organic matter decomposition and a more significant decrease in BOD values. In another study, dairy wastewater was used as substrate, and a higher removal efficiency of 88.02% BOD was achieved.^50^

Fig. 3a indicates that both *E. coli* and EM4 have an equivalent percentage BOD removal at a concentration of 20 percent of microorganisms. At such a high inoculum concentration, the degradation of biodegradable organic material is probably controlled more by the availability of substrates than by the type of microbe.^51^ The easily degradable portion of POME can be quickly digested by the two microbial systems leading to comparable BOD removal efficiencies. However, at lower inoculum levels, *E. coli* will have better performance, implying more effective use of the available substrates and quicker metabolic rate than the mixed microbial consortium in EM4.

Fig. 3b demonstrates the effect of microorganism concentration on the reduction of Chemical Oxygen Demand (COD) across all variations. A more substantial decrease in COD is associated with a higher microbial population within the substrate. As the number of microorganisms increases, more organic matter is degraded, resulting in a more pronounced reduction in COD. The abundance of microbes can be inferred from the corresponding biomass measurements obtained during the process. The reduction in COD levels during operation of the microbial fuel cell (MFC) reactor is attributed to microbial activity in the anode compartment, where organic compounds in the substrate are decomposed.^52^ The potential for energy generation in a microbial fuel cell (MFC) is directly proportional to the concentration of organic matter, as indicated by its chemical oxygen demand (COD). Higher COD levels provide a greater supply of oxidizable organic compounds for microbial metabolism, thereby improving subsequent electrical current production and the efficiency of electron transfer.^53^ MFC showed the best performance at COD 3325.0 mg L^−1^ with 89% removal efficiency and maximum power density of 339.41 mW m^−2^. Increasing the initial substrate concentration to 3825 mg L^−1^ decreased the efficiency of up to 72%, with a power density of 262.34 mW m^−2^.^54^ The greatest percentage reduction in COD levels was observed when *Escherichia coli* was added to the substrate, compared with the control (no microbial addition) and EM4 treatments. The most significant COD reduction occurred at an *E. coli* concentration of 20%, with a total decrease of 42%. The highest COD and BOD removal at 20% *E. coli* is an indication of a balance between the microbial population and the availability of the substrate. At high concentrations (25–30%), the removal efficiency was lower because of substrate limitation, increased microbial competition, generation of inhibitory metabolites and lower mass transfer efficiency which collectively inhibit the effective biodegradation.

### Electrical energy

**Fig. 4:**
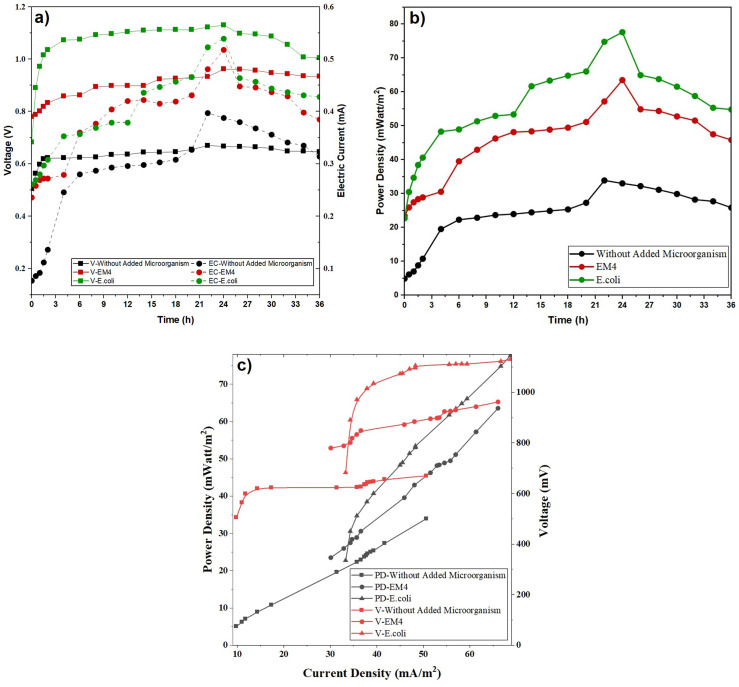
Effect of reaction time on voltage and electric current (a) and power density (b) and (c) and effect of current density on voltage-power density at different microorganism types and 15% concentration.

**Fig. 5:**
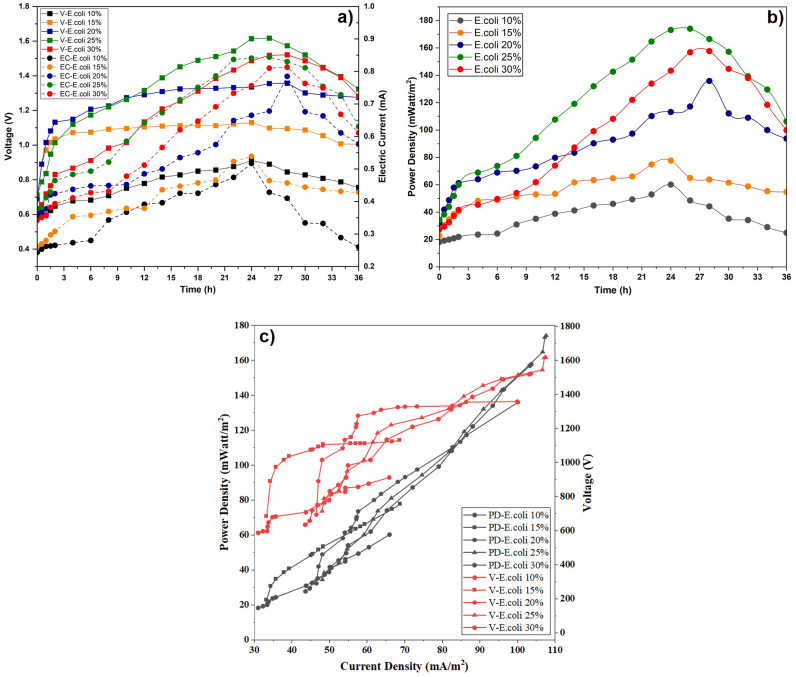
Effect of reaction time on voltage and electric current (a) and power density (b) and (c) effect of current density on voltage-power density at different *E. coli* concentrations.

The analysis of electrical energy in the microbial fuel cell (MFC) system was carried out in the presence and absence of *Escherichia coli* bacteria and EM4 added to POME waste. Voltage and electric current strength are produced in the MFC system due to ion movement, the difference in redox potential at the anode and cathode, and the chemical processes that occur at each of them.^55^ In this study, voltage (V) and current (mA) measurements were taken every 2 hours over the course of 36 hours of the MFC system. Fig. 4 shows the effect of reaction time on MFC system performance, as measured by voltage and current (Fig. 4a) and power density (Fig. 4b), for three conditions: without the addition of microorganisms, with EM4, and with *E. coli*. Fig. 5 presents the effect of varying *E. coli* concentration on MCC system performance, as indicated by changes in voltage and current (Fig. 5a) and power density (Fig. 5b) over reaction time.

As depicted in Fig. 4a and 5a, voltage (V) and current (mA) consistently increased over reaction time, ultimately reaching their optimal levels. In the early stages of measurement, the substrate was still sufficiently available to produce a relatively stable voltage (V) and current (mA). The increase in voltage and current is indicated due to the intensification of the electrochemical reaction rate in the operating MFC system. In addition, the accumulation of biomass on the anode electrode caused by the metabolic activity of microorganisms also increases the production of electrical energy.

Organic compounds in the substrate are oxidized by microorganisms, generating electrons that are subsequently transferred to the electrode. Therefore, optimal microbial growth and activity in the substrate result in increased voltage and current output.^56^ Over time, the concentration of metabolic intermediates and by-products decreases, reducing bioelectricity generation. This reduction continues until dynamic equilibrium is reached between microbial metabolic activity and the anodic oxidation rate. While the output of electrical energy stabilizes temporarily at this steady state, it subsequently declines due to the depletion of essential nutrients and electron donors in the substrate. This limits microbial respiration and electron transfer efficiency within the Microbial Fuel Cell (MFC) system.^6^

As shown in Fig. 2 and 4, the increase in biomass during the early operation period is correlated with the increase in current and power output, *i.e.* the growth of microbial is active, and the production of electrons is enhanced. However, at later stages, the subsequent increase of biomass is no longer accompanied by the subsequent increase of electrical output, which means that other factors, such as the limitation of substrates, the mass transfer, or the efficiency of electron transfer, become restrictive.

Measurements of voltage (V) and current (mA) indicated that the substrate variation supplemented with *E. coli* yielded the best electrical performance compared with the control treatment (without microbial addition) and the treatment with the EM4 microbial consortium. Among the tested concentrations of *E. coli*, the optimal value was achieved at 25% (v/v), reaching 1.617 V and 0.844 mA at the 26th hour. This indicates that *E. coli*, as a facultative anaerobe, has a better ability to utilize the organic substrate POME to produce electrons that are transferred to the anode.^57,58^ This difference can also be explained by the nature of *E. coli,* which is able to produce endogenous mediators (such as flavins and quinones), resulting in higher electron transfer efficiency.^59^

Palm oil mill effluent (POME) is a complicated mixture of organic compounds, including soluble carbohydrates, glycerol, volatile fatty acids, proteins, and the remaining lipids. Under the present MFC system, the readily biodegradable soluble sugars, glycerol and low-molecular-weight organic acids are preferentially oxidized at the anode by microbial metabolic reactions, which form the primary products of electrons, protons and carbon dioxide.^58^ More recalcitrant fractions, such as long-chain fatty acids, will presumably be converted more slowly and play a minor role in electricity generation in the short term.

The maximum cell voltage of 1.617 V that was measured in this study is the open-circuit voltage that was measured in batch conditions with a highly oxidative catholyte and does not imply electrochemical water splitting. A high voltage shows that the redox potential difference between microbial anode and permanganate-based cathode is high. Internal resistance and overpotentials when the cell is loaded make the cell voltage drop considerably, and is still far short of the thermodynamic threshold required to electrolyze water. In this way, the voltage obtained is consistent with the laws of electrochemical and does not indicate spontaneous water disintegration.

As shown in Fig. 5a, the increase in voltage and current from 10% up to 25% indicating a synergistic effect of the inoculum amount as consistent with the previous discussion regarding microorganism growth. A higher inoculum level accelerates the transition to the exponential phase, resulting in more electrons being produced per unit time.^60,61^ However, a literature also notes that an increase in excessive concentration can trigger a saturation effect due to limited substrate diffusion around the anode.^62^

Based on the present results, increasing *E. coli* concentration from 10 to 25% led to consistent improvements in biomass productivity and electrical output. Within the investigated range, 20% (v/v) represents the operational optimum in BOD removal and COD reduction, as it provided the highest overall performance under batch-operated conditions. Further increases in inoculum concentration beyond this level were not examined in this study. The additional increases in inoculum concentration could theoretically increase biodegradation; however, microbial kinetics theory and previous MFC studies indicate that excessive inoculum loading may lead to substrate limitation, intensified microbial competition, accumulation of inhibitory metabolites such as volatile fatty acids, and reduced mass and electron transfer efficiency at the anode surface.^63–65^

From a practical perspective, higher inoculum concentrations also increase microbial cultivation cost and operational complexity, which are critical factors for scalable MFC applications.^11,66^ Therefore, the optimum inoculum concentration in many MFC studies is defined as the level that balances performance enhancement and economic feasibility, rather than the maximum achievable concentration.^67^ Investigation of higher inoculum levels under continuous or fed-batch operation is proposed as future work.

Fig. 4b and 5b illustrate that the variation in power density varies with biomass level. When the microbial population increases, the electrical output generated by MFC system also increases.^68^ Over time, the electrical output decreases due to the gradual depletion of nutrients within the substrate, resulting from sustained microbial metabolic activity. The glucose concentration at the anode diminishes progressively, leading to a decline in the viable microbial cell population. Consequently, the metabolic rate decreases, causing a reduction in the electrical energy generated by the system. Initially, the power density was low due to bacterial adaptation to the new environment. It increased until the 28th hour, after which it declined due to reduced substrate availability. Decreasing power density in an MFC is mainly caused by limited supply of oxidized compounds, leading to a reduced bacterial population growth. Electricity is produced in an MFC due to the conversion of chemical energy into biodegradable organic substances.^69^

As indicated in Fig. 4a and 5a, the electrical output peaked at about 28 h and then decreased, which can be explained mainly by the depletion of easily biodegradable organic fractions in POME and the accumulation of metabolic by-products during batch operation. Even though POME contains intricate organic components, the short-term electrical response is primarily governed by the availability of readily oxidizable substrates rather than the total organic matter content. The current operation period of approximately 36 h was intended to evaluate the comparative performance of different microbial inocula under identical batch conditions rather than long-term operational stability. Prolonged operation times or repeated batch cycles would therefore be required to assess sustained performance and system stability, and are proposed as important directions for future research.

The highest power density was observed in the substrate variation supplemented with *E. coli*, compared to treatments without microbial addition and those with EM4 microbial. In Fig. 4b, the highest power density in the system with *E. coli* also confirms the role of this microorganism in accelerating organic degradation and producing more electrons per unit of substrate. Using a single bacterium with a high growth rate can improve MFC performance by 2–3 times compared to mixed microbial consortia, as interspecific competition can reduce electron transfer efficiency.^70,71^

In Fig. 5b, the highest power density was achieved with 25% *E. coli* (174.075 mW m^−2^ at 26 hours). This value is significantly higher than the 10%, 15% and 20% treatments, which each achieved only around 60–120 mW m^−2^. This trend aligns with previous findings that increasing the inoculum in POME-based MFCs can increase power density due to greater availability of active cells in the early phase of degradation.^72^ However, after reaching the peak, a decrease in power occurs due to the accumulation of organic acid metabolites, which can lower the local pH and inhibit electron transfer.^42,73^

Fig. 5c shows that increasing the *E. coli* concentration from 10 to 25% significantly increases the voltage and power density. This is due to the increased number of electroactive cells, which accelerate the oxidation of organic compounds and the transfer of electrons to the anode.^12^ A concentration of 25% remains the optimum condition, where biofilm formation and microbial metabolic activity occur effectively without any signs of inhibition due to substrate competition or diffusion limitations. The maximum electric production at 25% *E. coli* is an ideal proportion between the number of the microbes and the effectiveness of the electron transfer. At this state, the growth of biofilm and electrogenic activity is increased, and this improves the production and transfer of electrons. This was lower at the higher concentration (30%), due to the excessive thickness of the biofilm, mass transfer limitations, and increased internal resistance. This finding aligns with previous reports stating that increasing the inoculum to the optimum level can increase the current and power density in MFC systems.^74^Fig. 5c shows that the MFC system with POME + *E. coli* produced the highest electrical performance compared to EM4 and the control. This indicates that *E. coli* is more effective in utilizing POME organic compounds and transferring electrons to the electrode, both through direct transfer mechanisms and endogenous mediators.^57^ Conversely, EM4, as a microbial consortium, showed lower performance due to competition between microorganisms and metabolic pathways that do not fully contribute to electron transfer to the anode. Thus, these results confirm that the selection of specific microorganisms is more important than simply substrate complexity in determining MFC performance.

The power density (mW m^−2^) reported in this study is scaled to the projected surface area of the anode electrode and this definition has been explained to eliminate the ambiguity. No detailed electrochemical characterization of the microbial inocula, such as polarization curves and the determination of internal resistance, was done, and the main aim of this work was to offer a comparative evaluation of the microbial inocula performance under the same operating conditions, rather than to conduct an extensive evaluation of the electrochemical parameters of MFC. The values of power density reported therefore should not be taken as absolute measures of performance but as relative measures of electrical output. Additional electrochemical characterization of the electrochemical process would need future studies that include polarization analysis, internal resistance measurements, and normalization to both electrode area and reactor volume.

At higher inoculum concentrations (25% v/v), microbial activity promotes more efficient electron transfer toward the anode, resulting in enhanced electricity generation. In contrast, at 20% (v/v), a larger fraction of electrons is likely diverted to non-electrogenic pathways, such as biomass synthesis and fermentation, leading to greater COD and BOD removal but lower electrical output. This behavior can be attributed to the partitioning of electrons among competing metabolic pathways. This result highlights the distinct mechanisms governing organic matter biodegradation and electricity generation in MFC systems, which do not necessarily occur simultaneously at their optimum levels.

Table 3 shows the performance variations of POME-based microbial fuel cells (MFCs) using different microorganisms. Different types of microorganisms have been shown to affect both electrical energy output and pollutant degradation (COD and BOD) capabilities. Studies using *Lactobacillus bulgaricus* showed that this bacterium can generate a current of up to 3.35 mA at a voltage of 1.52 V. The reported power densities varied from 248.04 mW m^−2^ to 0.127 mW cm^−2^, depending on the reactor design and operating conditions.^11,24^ However, COD and BOD reduction were not reported in these studies. This is understandable because *Lactobacillus bulgaricus* is better known as a lactic acid bacterium that plays a role in fermentation, so its primary contribution to MFCs is the production of metabolites that support indirect electron transfer, rather than the complete degradation of complex organic compounds in solution.^75,76^

**Table 3:** Application of MFCs for electricity productivity from POME

Microorganism	Current generation	Power density	Voltage	COD reduction	BOD reduction	Reference
*Lactobacillus bulgaricus*	0.9640 mA	248.04 mW m^−2^	0.6760 V	7.2%	N/A	15
*Lactobacillus bulgaricus*	3.35 mA	0.127 mW cm^−2^	1.52 V	N/A	N/A	28
*Geobacter*	247 mA m^−2^	2.36 W m^−3^	N/A	N/A	N/A	74
*Shewanella* sp.	52 mA m^−2^	0.10 W m^−3^	N/A	N/A	N/A	74
*Galactomyces* sp.	215.56 ± 5.09 mA m^−2^	139.44 ± 6.56 mW cm^−2^	N/A	N/A	N/A	79
*Bacillus lichenformis*	N/A	0.18 ± 0.01 mW m^−3^	N/A	95.12 ± 0.15%	N/A	80
*Klebsiella variicola*	N/A	1.7 W m^−3^	N/A	43%	N/A	81
*Escherichia coli*	0.844 mA	174.075 mW m^−2^	1.617 V	42%	57%	This research

In contrast, *Geobacter* and *Shewanella* sp. is a pure electrogenic bacterium known to transfer electrons directly to electrodes *via* outer-separator cytochromes and nanowires.^59^ Therefore, both are often used in MFCs to improve electron transfer efficiency.^70,77^ The data in Table 3 shows that *Geobacter* can produce a current of 247 mA m^−2^ with a power density of 2.36 W m^−3^, while *Shewanella* sp. Produces 52 mA m^−2^ with a power density of 0.10 W m^−3^. However, similar to *Lactobacillus bulgaricus*, this study did not report COD or BOD reduction, so its contribution to wastewater treatment cannot be evaluated. This condition is common because research on electrogenic bacteria usually focuses on electron transfer mechanisms, rather than on the treatment of complex wastewater.^70,78^

The power density of this study is close to that of *Galactomyces* sp. (139.44 mW m^−2^; 215.56 mA m^−2^), although the study did not report voltage and COD/BOD reduction.^79^*Bacillus licheniformis* only recorded high COD reduction (95.12%) without current and voltage data, while *Klebsiella variicola* reported a power density of 1.7 W m^−3^ and 43% COD reduction, but without BOD data.^80,81^ The absence of COD/BOD data in those studies is generally due to their focus on electrogenic capacity and electron transfer efficiency, rather than complex waste degradation.

The results of this study using *E. coli* demonstrated distinct characteristics. *E. coli* produced a voltage of 1.357 V, a current of 0.785 mA, and a power density of 135.873 mW m^−2^. These values ​​are comparable and even higher than those of other studies, especially considering that the substrate used was highly complex, pure POME. More importantly, this study reported a 42% reduction in COD and 57% in BOD, findings not found in other studies. This indicates that in addition to functioning as an electrogenic catalyst, *E. coli* also plays a significant role in the degradation of organic matter in POME.

Physiologically, *E. coli* is a facultative anaerobic bacterium capable of rapid growth on organic-rich substrates, including POME. Its fermentative activity produces various intermediate metabolites (organic acids, hydrogen), which are then oxidized, generating electrons for transfer to the anode.^59^ The combination of rapid growth, abundant substrate availability, and adaptability in wastewater media explains why *E. coli* can generate high electrical output while significantly reducing the organic load of POME. Thus, compared to *Lactobacillus, Geobacter*, and *Shewanella*, the findings of this study demonstrate that *E. coli* is superior in practical applications: generating competitive electrical energy while contributing to waste remediation.

Regardless of the fact that *E. coli* is not a classical exoelectrogen, a number of studies have shown that it can play a role in electricity production in microbial fuel cells by mediated extracellular electron transfer (EET) processes.^82^ In more complex substrates like palm oil mill effluent (POME) *E. coli* is mostly involved in electron transfer indirectly through endogenous redox mediators, such as quinones and flavins, generated in intracellular metabolic events. Also, the oxidation of easily biodegradable organic compounds in POME by *E. coli* produces reduced metabolic intermediates that are able to engage in anodic electron transfer. The pathway is especially applicable in mixed and complex wastewater systems where microorganisms may not be in direct contact with the anode surface. The electrical output observed in the current study is therefore considered to be as a result of indirect electron transfer routes and not the direct outer-membrane cytochrome-mediated processes that are characteristic of *Geobacter* or *Shewanella* species.

Even though the mixed microbial consortia like EM4 are usually regarded as more resilient in the degradation of complex wastewater, their excellent treatment capacity may not always be reflected in increased electricity production. In the current research, EM4 would have probably favored hydrolysis and acidogenesis phases of POME degradation, but a large proportion of electrons could have been diverted to biomass production, fermentation products, or non-electrogenic metabolic activities. Conversely, the pure *E. coli* culture had a higher rate of substrate uptake and a more focused electron flow to anodic oxidation under the experimental conditions, which led to an increased electrical output. These results indicate that although microbial diversity is beneficial in the removal of organic matter, a less complex microbial system can be more useful in electricity production when cathodic constraints are reduced.^83^ This observation highlights the significance of matching the microbial selection with the main goal of the MFC system. Even with good organic matter decomposition, the proportion of electrons to be utilized in making anodic current might be low. Conversely, under the conditions of the study, the pure *E. coli* culture had a more directed substrate usage and electron flow toward anodic oxidation, which led to a greater electrical output. This observation indicates that the microbial community structure should be in line with the main purpose of the MFC system and not an indication of the inability of mixed consortia to treat wastewater.

## Conclusions

The optimal biomass concentration was recorded at 0.3118 g mL^−1^ at the 30th hour for the substrate supplemented with *Escherichia coli* at 25% (v/v), outperforming other substrate variations. The optimum incubation period was observed at the 26th hour, yielding a voltage of 1.617 V, a current of 0.844 mA, and a power density of 174.075 mW m^−2^ under the same treatment. However, the 20% (v/v) *E. coli* addition exhibited the greatest removal efficiencies, achieving 57% BOD and 42% COD reduction relative to other treatments. These outcomes underscore the relevance of tailored microbial systems in enhancing bio-electrochemical performance, paving the way for next-generation sustainable wastewater treatment technologies with integrated energy recovery potential.

## Conflicts of interest

The authors declare no competing financial or personal interests.

## Data Availability

All relevant data are included in this article. Additional information is available from the corresponding author upon request.
